# Effect of Psychological Intervention Combined with Family Cooperation on the Perioperative Quality of Life and Psychological States of Elderly Patients with Prostate Cancer Treated with Compound Kushen Injection

**DOI:** 10.1155/2021/2971644

**Published:** 2021-07-19

**Authors:** Jingyun Zhang, Caijian Li, Chengwei Fu, Jinkai Dong, Wei Guo, Qianqian Zhu

**Affiliations:** Department of Urology, The Fifth Medical Centre, Chinese PLA General Hospital, Beijing 100071, China

## Abstract

**Objective:**

The purpose of the study was to investigate the nursing effect of psychological intervention combined with family cooperation on elderly patients with prostate cancer treated with compound kushen injection and put forward effective suggestions.

**Methods:**

122 elderly patients with prostate cancer admitted to our hospital from June 2018 to June 2019 were selected and randomly divided into a control group (*n* = 61) and experimental group (*n* = 61). The patients in the control group received routine nursing intervention during the perioperative period, while the patients in the experimental group were treated with psychological intervention combined with family cooperation on the basis of routine nursing. The quality of life and psychological states of patients in the two groups were statistically analyzed.

**Results:**

The evaluation of psychological states at 24 hours before surgery and 24 hours before discharge in the experimental group was significantly better than that in the control group (*P* < 0.05), with statistical significance. On comparing the basic conditions between the two groups in the perioperative period, the length of hospitalization, length of catheter retention after surgery, and incidence of complications in the experimental group were all significantly better than those in the control group (*P* < 0.05), with statistical significance. The satisfaction of patients with the nursing process in both groups was recorded and statistically analyzed through questionnaires. The satisfaction with nursing process in the experimental group was significantly higher than that in the control group (*P* < 0.05), with statistical significance. The quality of life of the patients was followed up at three months after discharge. The quality of life of the experimental group was significantly better than that of the control group (*P* < 0.05), with statistical significance.

**Conclusion:**

Psychological intervention combined with family cooperation for the elderly patients with prostate cancer treated with compound kushen injection is beneficial to improve their psychological states, encourage them to face the disease in a more positive manner, effectively improve the quality of life after intervention, ensure the therapeutic effect during perioperative period, increase happiness index, and enhance their satisfaction with the nursing process, which is worthy of clinical application and popularization.

## 1. Introduction

According to relevant reports, with the aggravation of aging, people's life concept and quality of life have changed greatly, and the incidence of prostate cancer increases year by year [1–3]. Many studies have shown that compound kushen injection has a certain synergistic effect as an anticancer treatment, relieving pain, enhancing immunity, and stopping bleeding. It is an effective adjuvant drug for clinical treatment of prostate cancer and can significantly improve the clinical benefit of patients. To further ensure good prognosis in elderly patients with prostate cancer, scientific nursing measures must be combined. In elderly patients, the social adjustment capacity is decreased, and they are shy of the arrival symptoms of this disease, which results in huge psychological burdens. Therefore, most elderly patients with prostate cancer face stressful psychological reactions in the perioperative period, including depression and anxiety, which further adversely affect the therapeutic efficacy and physical recovery [4–6]. Based on this, in this study, psychological intervention combined with family cooperation was introduced to elderly patients with prostate cancer treated with compound kushen injection, and the patients' life quality and psychological states were evaluated, so as to further analyze the intervention's effect and provide data support for clinical research. The results of the study are summarized as follows.

## 2. Materials and Methods

### 2.1. General Information

One hundred and twenty-two elderly patients with prostate cancer admitted to our hospital from June 2018 to June 2019 were selected and randomly divided into control group (*n* = 61) and experimental group (*n* = 61), aging from 55 to 81 years, with the average age of 69.37 years. There were no statistical differences in the comparison of general data such as age in both groups (*P* > 0.05), which was comparable, and the comparison of general data between the two groups is detailed in Table 1.

### 2.2. Inclusion Criteria

The inclusion criteria are as follows. (1) Patients met the clinical diagnostic criteria for prostate cancer according to the *Diagnostic Criteria for Prostate Cancer*. (2) All patients received compound kushen injection as an adjuvant therapy. (3) Patients had complete clinical records. (4) This study was approved by the Hospital Ethics Committee, and the patients and their families were informed of the purpose and process of this study and signed the informed consent.

### 2.3. Exclusion Criteria

The exclusion criteria are as follows. (1) Patients had other malignant and severe diseases. (2) Patients had other acute or long-term urinary system diseases. (3) Patients had cognitive impairment, such as mental disorders, or refused to cooperate with the study. (4) Patients had incomplete clinical data.

### 2.4. Methods

Patients in the control group were treated with the routine clinical nursing. According to the diagnosis results, preoperative examination and preparation were performed. The medical staff actively participated in the process of formulating the patients' surgical plans, reasonably evaluated patients' conditions, and made a scientific nursing intervention program. Besides, during the surgery, the medical staff cooperated with surgeon to complete the surgical process and paid close attention to the changes of patients' vital signs. After the surgery, routine index examination for patients was performed, and patients' dietary management and reasonable mix of nutrition were also taken into consideration [7–9].

Patients in the experimental group received psychological intervention combined with family cooperation. After patients' admission to hospital, psychological intervention was conducted on the basis of routine nursing by keeping an eye out for patient's psychological changes. According to the patients' conditions, the medical staff informed patients and their family members of the cause of the disease, specific conditions, and treatment plan of prostate cancer in a gentle way. Additionally, the medical staff also explained the relevant knowledge of prostate cancer in detail, thoroughly understood the concerns of patients and their family members, and paid attention to emotional comfort. By describing similar successful cases, the medical staff increased the confidence of patients and their families and eliminated the fear before the surgery. After the surgery, patients and their families were informed of the results of the surgery in time and were encouraged to cooperate closely with the treatment. The patients' family members were told to conduct good supervision and support work to further increase the patients' confidence [10–12]. The nursing staff and family members cooperated with each other to perform the routine nursing and introduce psychological intervention during the perioperative period and observed the psychological changes of the patients acutely, so as to increase the patients' sense of security [13].

### 2.5. Observation Indexes

#### 2.5.1. Scores of Self-Rating Anxiety Scale (SAS) and Self-Rating Depression Scale (SDS) in Both Groups

Self-rating anxiety scale (SAS) and self-rating depression scale (SDS) were adopted to evaluate the psychological states of patients at 24 hours after admission, 24 hours before the surgery, and 24 hours before discharge, with the total score of 100 points, and higher scores indicated patients' severer psychological conditions.

#### 2.5.2. Basic Conditions in Both Groups during the Perioperative Period

The basic conditions of the patients in the two groups during the perioperative period were recorded in detail, including the length of the operation, bladder irrigation time, length of catheter retention after surgery, length of hospitalization, and incidence of complications.

#### 2.5.3. Nursing Satisfaction in Both Groups

The nursing satisfaction of patients in the two groups was investigated with a self-made questionnaire from the hospital, which mainly included the nurses' attitudes, quality of work, professional degree, and so on, with the total score of 100 points. A score above 90 points indicated “very satisfied,” a score of 70–90 points indicated “basically satisfied,” and a score below 70 points indicated “unsatisfied.” Total nursing satisfaction = basically satisfied + very satisfied.

#### 2.5.4. Statistical Analysis of the Postoperative Quality of Life in Both Groups

The quality of life of the patients after surgery was evaluated by three evaluation indexes, international prostate symptom score, rehabilitation knowledge evaluation, and comprehensive evaluation of quality of life, so as to further record and analyze the patients' physical recovery, family emotion environment, body function, social skill and psychological states.

### 2.6. Statistical Treatment

The data obtained in this study were statistically analyzed and processed by SPSS20.0 software. Measurement data were expressed by ($\bar{x}$ ± *s*) and tested by *t*-test. Enumeration data were expressed as [*n* (%)] and tested by *X*^2^ test. The differences had a statistical significance when *P* < 0.05.

## 3. Results

### 3.1. Comparison of the SAS and SDS Scores between the Two Groups

The SAS and SDS scores of patients at 24 hours after admission, 24 hours before the surgery, and 24 hours before discharge were compared and analyzed, and the results were as follows.

**Figure 1:**
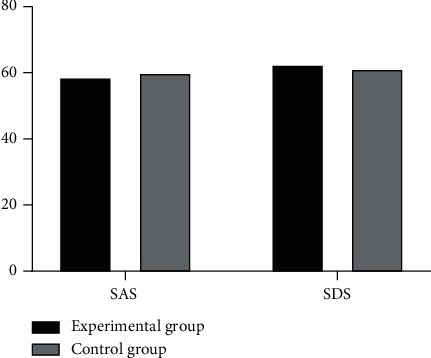
Comparison of SAS and SDS scores between the two groups at 24 hours after admission (*n* = 61). The abscissa represents SAS and SDS, while the ordinate represents score. The SAS and SDS scores in the experimental group at 24 hours after admission were 58.72 ± 6.08 and 62.56 ± 7.91, respectively. The SAS and SDS scores in the control group at 24 hours after admission were 60.02 ± 6.32 and 61.31 ± 8.02, respectively. There were no significant differences in SAS and SDS scores between the two groups at 24 hours after admission (*t* = 1.7578, *P*=0.2493).

There were no significant differences in SAS and SDS scores of patients between the two groups at 24 hours after admission, as shown in Figure 1.

**Figure 2:**
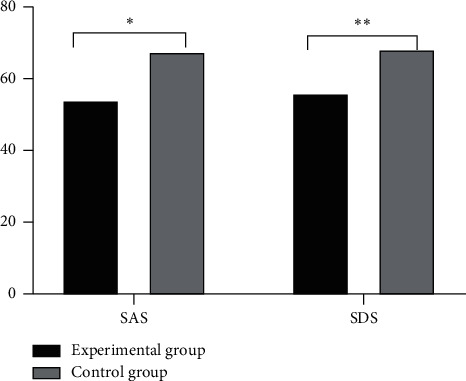
Comparison of SAS and SDS scores between the two groups at 24 hours before surgery (*n* = 61). The abscissa represents SAS and SDS, while the ordinate represents score. The SAS and SDS scores of the experimental group at 24 hours before surgery were 54.11 ± 5.92 and 56.12 ± 6.46, respectively. The SAS and SDS scores of the control group at 24 hours before surgery were 67.63 ± 8.67 and 68.37 ± 7.13, respectively. ^*∗*^indicates that the SAS score of the experimental group was significantly better than that of the control group (*t* = 10.0582, *P* ≤ 0.001). ^*∗∗*^indicates that the SDS score of the experimental group was significantly better than that of the control group (*t* = 9.9442, *P* ≤ 0.001).

The SAS and SDS scores of the experimental group at 24 hours before the surgery were significantly better than those of the control group, as shown in Figure 2.

**Figure 3:**
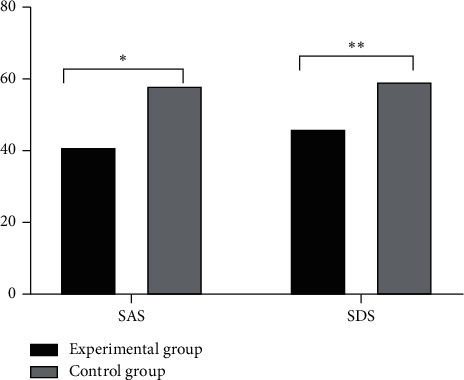
Comparison of SAS and SDS scores between the two groups at 24 hours before discharge (*n* = 61). The abscissa represents SAS and SDS, while the ordinate represents score. The SAS and SDS scores of the experimental group at 24 hours before discharge were 41.26 ± 5.72 and 46.31 ± 6.01, respectively. The SAS and SDS scores of the control group at 24 hours before discharge were 58.33 ± 6.08 and 59.48 ± 7.11, respectively. ^*∗*^indicates that the SAS score of the experimental group was significantly better than that of the control group (*t* = 15.9709, *P* ≤ 0.001). ^*∗∗*^indicates that the SDS score in the experimental group was significantly better than that in the control group (*t* = 11.0487, *P* ≤ 0.001).

The SAS and SDS scores of the experimental group at 24 hours before discharge were significantly better than those of the control group, as shown in Figure 3.

### 3.2. Comparison of the Basic Conditions between the Two Groups in the Perioperative Period

According to the comparison between the basic conditions of the two groups in perioperative period, it is concluded that the length of hospitalization, length of catheter retention, and incidence of complications in the experimental group were significantly better than those in the control group (*P* < 0.05), with statistically significant differences, as shown in Table 2.

### 3.3. Comparison of the Nursing Satisfaction between the Two Groups

The nursing satisfaction of the elderly patients with prostate cancer in the two groups was statistically analyzed in the form of questionnaires. According to the results, the total satisfaction of nursing in the experimental group was significantly higher than that in the control group (*P* < 0.05), with statistically significant differences, as shown in Table 3.

### 3.4. Comparison of the Quality of Life between the Two Groups after Surgery

**Figure 4:**
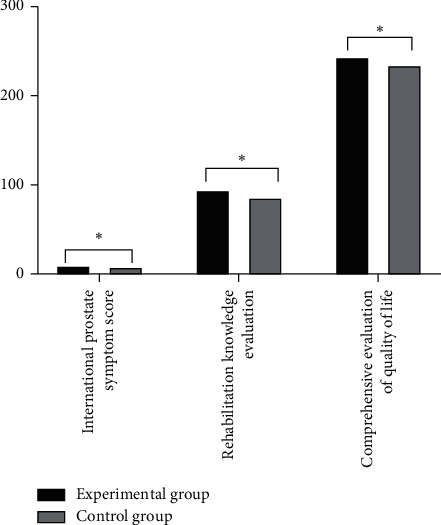
Comparison of the postoperative quality of life scores between the two groups (*n* = 61, $\bar{x}$ ± *s*). The abscissa represents international prostate symptom score, rehabilitation knowledge evaluation, and comprehensive evaluation of quality of life, while the ordinate represents score. The international prostate symptom score, rehabilitation knowledge evaluation, and comprehensive evaluation of quality of life in the experimental group were 9.67 ± 3.71, 94.47 ± 3.12, and 243.51 ± 9.76, respectively. The international prostate symptom score, rehabilitation knowledge evaluation, and comprehensive evaluation of quality of life in the control group were 8.21 ± 3.02, 85.98 ± 3.01, and 234.72 ± 10.49, respectively. ^*∗*^indicates that there were significant differences in the international prostate symptom score, rehabilitation knowledge evaluation, and comprehensive evaluation of quality of life between the two groups (*t* = 2.3837, 15.2953, 4.7914, *P* < 0.05).

The quality of life of the two groups was investigated and evaluated at three months after surgery, as shown in Figure 4.

## 4. Discussion

Prostate cancer is actually a relatively slow-growing tumor, and thus, patients may not be aware of the early-stage tumor growth. However, for patients with advanced cancer, the deterioration of the disease not only seriously impairs patients' physical function but also adversely affects patients' psychological states and quality of life [14–16]. With the development and progress of modern medical technology, the cure rate of the disease is increasing, and most patients with prostate cancer will receive compound kushen injection for comprehensive endocrine treatment in order to control the number of tumor cells, improve clinical symptoms, enhance the quality of life, and prolongsurvival time. However, the psychological changes of patients are often ignored in practice, and adverse psychological emotions or inadequate family support will also have a negative impact on the treatment effect of patients, leading to unsatisfactory results of compound kushen injection [17–20]. With the advancement of medicine, more attention has been paid to the psychological recovery of patients, especially in elderly patients with prostate cancer. Because the treatment method mainly targets the patients' special parts through the urinary system surgery, patients are prone to greater psychological burdens. Therefore, a scientific psychological intervention mode is more conducive to the postoperative rehabilitation of patients [21–24].

In this study, it was found that the evaluation of the psychological states of the experimental group was significantly better than that of the control group at 24 hours before surgery and 24 hours before discharge. On comparing the basic situations between the two groups during the perioperative period, the length of hospitalization, length of catheter retention after surgery, and incidence of complications in the experimental group were significantly better than those in the control group. According to the satisfaction in the two groups evaluated by the questionnaires, the satisfaction of the patients with the nursing process showed that the total satisfaction of nursing in the experimental group was significantly higher than that in the control group, and the quality of life score in the experimental group at three months after discharge was also significantly better than that in the control group. From the study results, it can be easily concluded that, due to the specificity of the surgery, the scores of preoperative psychological states in the two groups are high. When facing the upcoming surgery, the patients are easily restless, anxious, and stressed. However, the evaluation of patients' psychological states in the experimental group with the preoperative psychological intervention was significantly better than that in the control group, indicating that the psychological intervention combined with family cooperation can better help patients with good psychological construction, reduce the psychological stress of patients, and promote the postoperative rehabilitation of elderly patients with prostate cancer. The results of this study were similar to those of Beardo et al. [25], whose study showed that elderly patients with cancer were more prone to emotional fluctuations, and patients had a better therapeutic effect under the supervision of nursing staff during hospital treatment. It also pointed out that many elderly patients after discharge were subject to decreased medication compliance due to the various effects of mood and life, thus leading to the occurrence of adverse symptoms, slow recovery of physical health, or treatment failure in severe cases. However, perioperative psychological intervention can effectively improve the patients' psychological states and enhance their treatment confidence.

In conclusion, psychological intervention combined with family support and adequate nursing care in the perioperative period for elderly patients with prostate cancer is more conducive to enhancing the patients' confidence in overcoming the disease. Both family support and care from the nursing staff can bring more psychological support to the elderly patients, which is easier for the patients to accept their conditions and improve their life quality, especially in the recovery process after surgery.

## Figures and Tables

**Table 1:** General information of elderly patients with prostate cancer in both groups (n = 61).

	Experimental group	Control group	*T* or *X*^2^	*P*
Age (years)			1.1781	0.2411
	62.4 ± 5.63	63.6 ± 5.62		

Education			0.1529	0.696
Below elementary education	20 (32.79%)	18 (29.51%)		
Elementary education and above	41 (67.21%)	43 (70.49%)		

Past medical history			0.0331	0.856
Hypertension	16 (26.23%)	14 (22.95%)		
Diabetes	12 (19.67%)	13 (21.31%)		
None	33 (54.09%)	34 (55.74%)		

Smoking			0.0575	0.810
Yes	50 (81.97%)	51 (83.61%)		
No	11 (18.03%)	10 (16.39%)		

Drinking			0.0503	0.823
Yes	49 (80.33%)	48 (78.69%)		
No	12 (19.67%)	13 (21.31%)		

Residence			0.1488	0.700
Urban area	42 (68.85%)	40 (65.57%)		
Rural area	19 (31.15%)	21 (34.43%)		

**Table 2:** Comparison of the basic conditions between the two groups in the perioperative period (*n* = 61, $\bar{x}$ ± *s*).

Group	Length of operation (min)	Bladder irrigation time (h)	Length of hospitalization (d)	Length of catheter retention after surgery (d)	Incidence of complications
Experimental group	60.33 ± 12.47	23.56 ± 7.04	3.77 ± 0.86	2.74 ± 0.78	18.03% (11/61)
Control group	59.62 ± 12.74	22.71 ± 7.51	5.71 ± 1.06	3.90 ± 1.21	34.43% (21/61)
*t*/*X*^2^	0.3111	0.6449	11.1004	6.2933	4.2361
*P*	0.7563	0.5202	*P* ≤ 0.001	*P* ≤ 0.001	0.040

**Table 3:** Comparison of the nursing satisfaction between the two groups (*n* = 61).

Group	Unsatisfied	Basically satisfied	Very satisfied	Total satisfaction
Experimental group	4.92% (3/61)	29.51% (18/61)	65.57% (40/61)	95.08% (58/61)
Control group	22.95% (14/61)	37.7% (23/61)	39.34% (24/61)	77.05% (47/61)
*X* ^2^				8.2700
*P*				0.004

## Data Availability

All primary data are available from the corresponding author upon a reasonable request.
